# Realizing High-Performance Vacuum-Deposited Inverted α‑FAPbI_3_ Perovskite Solar Cells through Saturated-Humidity Annealing

**DOI:** 10.1021/acsami.5c24183

**Published:** 2026-03-18

**Authors:** Yun-Sheng Jheng, Cheng-Yueh Chen, Pei-En Jan, Hung-Ming Chen, Hao-Cheng Lin, Ping-Hsun Tsai, Chia-Feng Li, Yu-Ching Huang, Minh Anh Truong, Atsushi Wakamiya, Hao-Wu Lin

**Affiliations:** † Department of Materials Science and Engineering, 34881 National Tsing Hua University, Hsinchu 30013, Taiwan; ‡ Department of Materials Engineering, 56082 Ming Chi University of Technology, New Taipei City 24301, Taiwan; § Institute for Chemical Research, 26337 Kyoto University, Gokasho, Uji, Kyoto 611-0011, Japan; ∥ Research Center for Critical Issues, Academia Sinica, Tainan 711, Taiwan

**Keywords:** perovskite solar cells, vacuum deposition, FAPbI_3_, saturated-humidity, environmental light harvesting

## Abstract

Achieving high-efficiency perovskite solar cells (PSCs) typically necessitates postdeposition annealing under stringent environmental controls, such as low relative humidity or inert atmospheres. These demanding conditions significantly increase the complexity and cost of device fabrication. Therefore, developing an annealing strategy for obtaining high-quality, phase-pure α-FAPbI_3_ perovskite in ambient or high-humidity environments is critically important. Here, we demonstrate that coevaporated FAPbI_3_ perovskite active layers annealed in a saturated-humid environment exhibit pure α-phase and thus markedly superior device performance compared to those annealed in normal air or an inert atmosphere. The resulting preferred crystal orientation, superior carrier transport properties, and retarded charge carrier recombination collectively enable an excellent power conversion efficiency (PCE) of up to 21.3% in vacuum-deposited inverted PSCs. Additionally, these devices also deliver excellent performance under indoor environmental light-harvesting, achieving PCEs of 34.9% and 36.7% under 1000 lx illumination from 6500 K and 3000 K fluorescent light sources, respectively.

## Graphical abstract

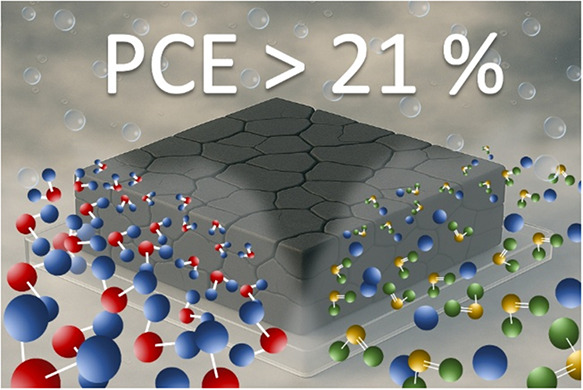

## Introduction

Metallic halide perovskites have garnered significant attention in recent years due to their exceptional material properties, including tunable bandgap,^1^ long carrier diffusion lengths,^2^ and high absorption coefficients,^3^ such characteristics render them highly competitive for the development of next-generation photovoltaic technologies.^4–9^ Over the past decade, the power conversion efficiencies (PCEs) of perovskite solar cells (PSCs) have increased rapidly, with certified values surpassing 27% and now exceeding well-developed thin film technologies such as cadmium telluride (CdTe) and copper indium gallium selenide (CIGS). Although the majority of high-efficiency PSCs reported to date have been fabricated using solution-based methods, these techniques present several limitations, including extensive reliance on toxic solvents and difficulties in achieving uniform, large-area deposition. These factors raise concerns regarding their scalability and commercial viability.^10,11^ In contrast, vacuum deposition techniques present several notable advantages, including solvent-free processing, conformal film formation,^12^ and excellent compatibility with large-scale manufacturing.^13–15^ These attributes enable the fabrication of perovskite modules with high uniformity over large areas. Additionally, vacuum evaporation techniques are inherently compatible with existing semiconductor manufacturing infrastructure, thereby facilitating seamless integration into current industrial production lines.^11^ Beyond this, vacuum-based processes are particularly well-suited for the development of perovskite/silicon tandem solar cells,^16–18^ owing to their precise thickness control and material versatility.^11,18–20^

Among various vacuum-based techniques, coevaporation stands as a common and mature fabrication method.^21^ This approach facilitates the simultaneous vaporization of lead halide and organic ammonium salt under high vacuum, enabling the formation of compositionally homogeneous perovskite films. In 2013, Snaith et al, reported the first demonstration of coevaporated PSCs, wherein MAPbI_*x*_Cl_3–*x*_ absorber layers achieved a PCE exceeding 15% through precise control of PbCl_2_ and MAI deposition rates, monitored via quartz crystal microbalances.^20^ More recently, formamidinium iodide (FAI)-based perovskites have garnered significant interest as superior alternatives to their methylammonium iodide (MAI)- based counterparts. This preference stems from their enhanced thermal stability, reduced ion migration and phase segregation tendencies, and more favorable optoelectronic properties. Building on this, in 2017, Johnston et al. successfully achieved coevaporated FAPbI_3_ PSCs with a PCE of 15.8% and uniform large-area (64 cm^2^) FAPbI_3_ films.^22^ The sustained interest in coevaporation has continuously driven advancements in material complexity, including multication and mixed-halide systems.^23,24^ Currently, numerous novel processing techniques, such as rapid deposition, the use of molten salt precursors, and transport layer engineering, have also developed rapidly.^25^ Many novel ideas have been applied to coevaporated PSC research; however, relatively few studies have investigated the fundamental aspects, specifically the impact of moisture exposure during annealing on perovskite formation. Notably, perovskite materials exhibit pronounced moisture sensitivity, which poses a significant challenge during thin-film fabrication.^26,27^ Due to the high susceptibility of solvent–precursor intermediates to moisture, which often induces uncontrolled nucleation processes and compromises film quality, most high-efficiency solution-processed FAPbI_3_ PSCs necessitate postdeposition annealing under controlled conditions, typically at relative humidity (RH) of around 30% or lower, or in inert atmospheres such as Ar or N_2_.^28–31^ These stringent environmental requirements considerably increase the complexity and cost of the fabrication process. In contrast, the solvent-free vacuum deposition process eliminates the formation of such sensitive intermediates, thereby preventing moisture-induced disruption during the crystallization process and allowing water vapor to exert a beneficial influence during postannealing. Therefore, optimizing annealing methods that facilitate α-phase stabilization and high-quality vacuum-processed α-FAPbI_3_ thin film formation under ambient or high humidity atmospheres is critically important. While the influence of moisture on MAPbI_3_ has been extensively investigated,^32,33^ the role of humidity in the annealing of vacuum-deposited FAPbI_3_ remains largely unexplored. To the best of our knowledge, no comprehensive studies have yet addressed the impact of moisture during the annealing of coevaporated FAPbI_3_ films, underscoring this as a potentially impactful research area.

P-i-n (inverted) structure PSCs offer distinct advantages over their n–i–p counterparts. These include reduced interfacial trap densities, which effectively mitigate hysteresis, and a higher suitability for low-temperature processing, enabling their integration with flexible substrates and large-scale production.^34^ Notably, materials commonly utilized in conventional n–i–p device architectures, such as expensive hole-transporting layers (HTLs) (e.g., spiro-OMeTAD) and electrodes (e.g., Au), typically lead to higher fabrication costs compared to the materials employed in inverted PSCs (e.g., C_60_, PEDOT:PSS as carrier-transporting layers; Ag as electrode).^35^ Moreover, the integration of self-assembled monolayers (SAMs) as HTLs has become increasingly prevalent in inverted device configurations.^36,37^ Building upon insights from these studies, our work also investigates the impact of different SAMs employed as HTLs on the performance of inverted PSCs. These advantages collectively highlight the promising potential of inverted PSCs, particularly when fabricated via vacuum deposition. However, in contrast to solution-processed PSCs—for which high device efficiencies have been demonstrated in both inverted and n-i-p architectures, the majority of high-efficiency vacuum-processed PSCs reports employ the conventional n-i-p architecture.^25,38–40^ Studies on efficient vacuum-sublimed PSCs with inverted configurations remain limited, with the highest reported PCE to date being 23.0%, a value still below that of n-i-p devices.^41^ This discrepancy may be attributed to the observation that perovskite films deposited on crystalline SnO_2_ or TiO_2_ exhibit superior crystallinity, surface morphology, and lower defect density compared to those grown on the amorphous HTL, which consequently leads to differences in device efficiencies between the two architectures.^22,25,38–40,42–44^ Considering the high material costs associated with conventional n-i-p device architectures, as well as the growing demand for compatibility with future technologies such as tandem perovskite/silicon solar cells,^16–18^ the investigations of achieving high-efficiency inverted PSCs becomes a key research direction for the future of photovoltaic technology.

In this work, we investigate the effect of annealing humidity environment on the performance of vacuum-deposited PSCs. To our surprise, we found that the one with the perovskite active layer annealed in a saturated-humid (RH 99%) environment exhibited significantly superior PCE compared to those annealed in normal air (RH 55%) and an inert environment (N_2_). To elucidate the underlying mechanisms, we systematically perform a series of characterizations on coevaporated FAPbI_3_ films annealed under varying humidity. These analyses included scanning electron microscopy (SEM), atomic force microscopy (AFM), X-ray diffraction (XRD), grazing-incidence wide-angle X-ray scattering (GIWAXS), steady-state photoluminescence (PL) and time-resolved photoluminescence (TRPL) measurements. Our findings confirm that high humidity during annealing has a positive impact on the resulting perovskite quality. We demonstrated a significant correlation between the high-quality, phase-pure α-FAPbI_3_ thin films and annealing humidity, with optimal results achieved through annealing at RH of 99%. The champion device, annealed in a saturated-humidity environment, achieved a PCE of 19.5% using 3PATAT-C3 as the HTL. By replacing 3PATAT-C3 with (4-(3,6-Dimethyl-9*H*-carbazol-9-yl)­butyl)­phosphonic acid (Me-4PACz), the PCE was further improved to 21.3%, accompanied by a notably enhanced open-circuit voltage (*V*_OC_) of 1.10 V, demonstrating competitive performance of vacuum-deposited pure FAPbI_3_ PSCs in the inverted structure.^45,46^ Furthermore, PCEs of 34.9% and 36.7% were achieved under 1000 lx illumination of 6500K and 3000K artificial light sources, respectively, highlighting the strong potential of vacuum-deposited FAPbI_3_ PSCs not only for 1-sun intensive light harvesting but also for dim-light applications.

## Results and Discussion

Figure 1a displays a schematic illustration of the device fabrication chamber. In this work, we utilized a dual-source coevaporation process to fabricate FAPbI_3_ active layers by placing PbI_2_ and FAI into separate crucibles and coevaporating them simultaneously. The deposition rates of individual materials were precisely monitored via separated quartz crystal monitors (QCMs), thereby ensuring accurate stoichiometric ratio of the perovskite precursors. Notably, the vacuum chamber employed for this study is the same as that utilized in our previous publications on MAPbI_3_ PSCs, demonstrating a general applicability of vapor deposition technique within a common fabrication facility.^6–8^ After deposition, the perovskite precursor films were transferred into a custom-designed apparatus for postannealing, as illustrated in Figure 1b. Humidity was controlled by placing a beaker filled with water on a hot plate to generate water vapor. The apparatus was equipped with both an ambient hygrometer and an additional sensor positioned near the substrate to monitor local temperature and relative humidity during annealing. Three distinct annealing conditions were established: a nitrogen-filled glovebox (N_2_), ambient air (RH 55%), and a water vapor-saturated environment (RH 99%). Notably, the RH 99% postannealing condition can be reproducibly achieved using a simple sealed configuration, without requiring stringent dry-room or inert-atmosphere control. In contrast, low-humidity processing (RH < 30%) generally necessitates dedicated environmental control infrastructure, whereas a saturated RH 99% environment can be achieved without specialized equipment. Operating near the saturation limit further provides a robust processing window that is less sensitive to minor fluctuations in ambient laboratory humidity. Figure S1 (Supporting Information) presents optical images of the experimental setup for achieving the RH 99% postannealing condition. Photographs of the perovskite films annealed under different conditions are shown in Figure 1c. The films annealed at RH 55% and RH 99% conditions exhibited smooth, mirror-like appearances, whereas those annealed in N_2_ displayed a visible surface nonuniformity (highlighted by red circular markings). To further evaluate the scalability of the proposed approach, uniform vacuum-deposited perovskite films and corresponding devices with areas up to 25 cm^2^ and 4.5 cm^2^, respectively, were successfully fabricated using the RH 99% annealing process, as shown in Figure S2 (Supporting Information), demonstrating the potential of this RH 99% annealing method for large-area perovskite film and device fabrication.

**Figure 1:**
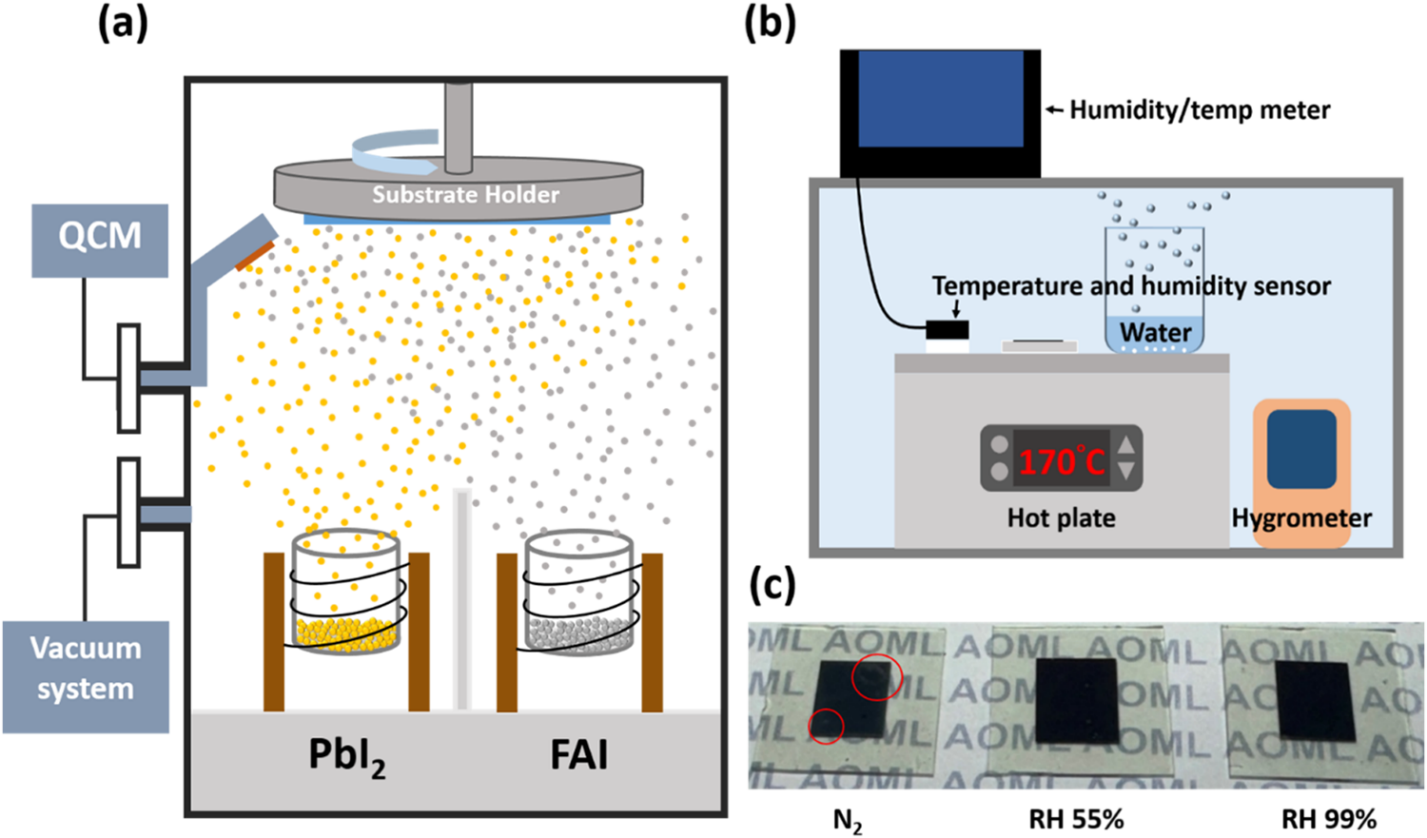
(a) Schematic illustration of the perovskite reaction chamber and dual-source coevaporation process. (b) Illustration of a custom-designed apparatus for postannealing of perovskite. (c) Photographs of the perovskite thin films annealed under various conditions.

As presented in Figure S3 (Supporting Information), The pristine (as-deposited) film exhibited a fine-grained microstructure. To investigate the impact of annealing conditions on the surface morphology of perovskite thin films, we conducted SEM and AFM analyses on the annealed coevaporated perovskite thin films. SEM and AFM images of perovskite films annealed under different conditions are presented in Figures 2 and S5 (Supporting Information). As shown in top-view SEM images (Figure 2a–c), the perovskite thin film annealed in N_2_ contained both large and small grains, whereas the thin films annealed at RH 55% and RH 99% displayed more homogeneous morphologies. This trend is further confirmed by the quantitative statistical results presented in Figure S4 (Supporting Information) and Table S1. The narrowing of the grain size distribution under elevated humidity is quantitatively reflected by the progressive decrease in the coefficient of variation (CV = σ/μ) from 0.58 to 0.53. Concurrently, the average grain size showed a progressive increase from 0.28 μm (N_2_) to 0.31 μm (RH 55%) and reached 0.36 μm at RH 99%. Consequently, the film annealed at saturated-humidity exhibited the coarsest grain structure among the three annealing conditions. Smaller grains are generally associated with several drawbacks, including an increased number of grain boundaries, which can promote nonradiative recombination and lead to reduced *V*_OC_ and fill factor (FF).^7^ Moreover, the limited carrier diffusion length in small-grain films can result in a lower short-circuit current density (*J*_SC_), ultimately degrading the overall device PCE.^47^

**Figure 2:**
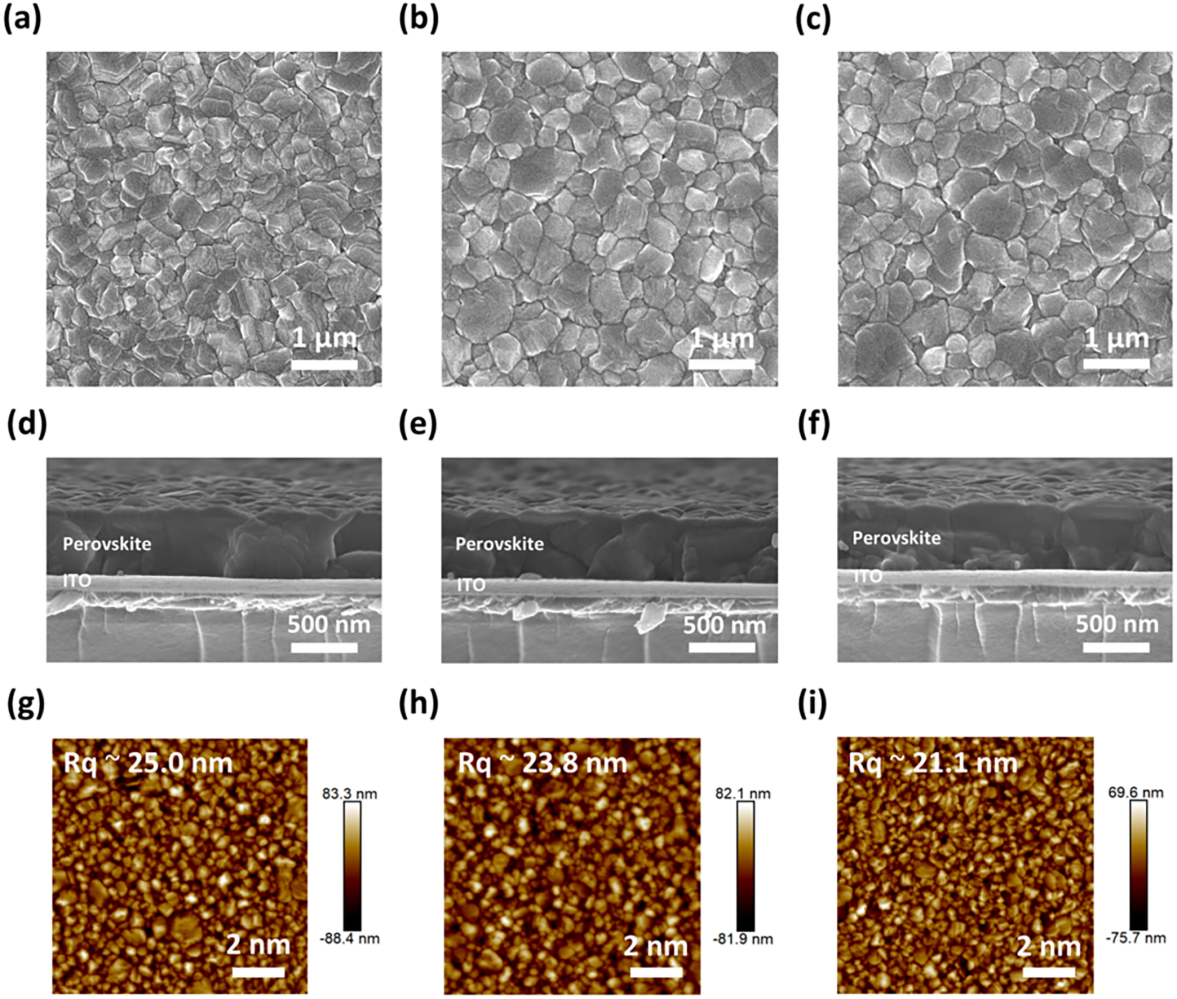
(a–c) Top-view SEM images, (d–f) cross-sectional SEM images, and (g–i) AFM topography images of perovskite thin films annealed under different conditions: (a, d, g) N_2_, (b, e, h) RH 55%, and (c, f, i) RH 99%.

Cross-sectional SEM images (Figure 2d–f) reveal that the final thicknesses of the perovskite layers were approximately 500 nm. Notably, the film annealed at RH 99% (Figure 2f) displayed a vertically aligned columnar grain structure. Such a structure is widely recognized as an indication of high efficiency PSCs, as it facilitates efficient charge carrier transport in the vertical direction, thereby minimizing recombination at grain boundaries and enhancing extraction to adjacent charge-transporting layers.^48^

Furthermore, we analyzed the surface roughness of the perovskite films using AFM, as shown in Figures 2g–i and S5 (Supporting Information). The root-mean-square roughness (*R*_q_) of the perovskite thin films decreases with increasing annealing humidity. Specifically, the film annealed at RH 99% showed the lowest *R*_q_ value of approximately 21.1 nm. This observation highlights the smooth surface morphology of the coevaporated perovskite films, achieved without the need for dynamic techniques such as antisolvent dripping employed in solution fabrication. The smooth morphology of the perovskite film also suggests that a thin upper electron-transporting layer is sufficient to efficiently inhibit direct contact between the perovskite and the electrode.

The absorption spectra of the pristine sample and perovskite thin films annealed under different conditions is presented in Figure S6 (Supporting Information). The pristine film exhibited substantially lower absorbance across the visible region, which is attributed to the dominant presence of the δ-phase. In contrast, all postannealed films showed markedly enhanced absorption without any additional absorption features from secondary phases, indicating complete conversion to the pure α-FAPbI_3_. Notably, the absorption edges of all perovskite thin films, regardless of the annealing environment, were consistently located near 810 nm, which is a typical characteristic of FAPbI_3_ perovskites. The corresponding bandgap values derived from the absorption edge are shown in Figure S7 (Supporting Information). Specifically, the perovskite films annealed in N_2_, at RH 55%, and at RH 99% exhibit bandgaps of 1.532, 1.523, and 1.533 eV, respectively. These results indicate that the annealing environment has no significant impact on the bandgap of the FAPbI_3_ perovskite. Among the fully annealed perovskite films, subtle differences in optical absorbance are still observed. These minor variations are reasonably associated with humidity-dependent microstructural differences, as higher annealing humidity promotes larger and more uniformly distributed grains, thereby facilitating improved grain coalescence and reduced nonabsorbing grain-boundary regions.^49^

The phase evolution of FAPbI_3_ thin films under varied annealing atmospheres was systematically investigated via XRD, as presented in Figure 3a–c. The initial state of the pristine film was characterized by a dominant diffraction peak at 11.7°, confirming the substantial presence of the δ-phase FAPbI_3_ with an intensity significantly surpassing that of the desired α-phase FAPbI_3_, in good agreement with the absorption spectra presented in Figure S6 (Supporting Information). Upon thermal annealing, a complete phase transition from the δ-phase to the α-phase was successfully achieved across all three annealing conditions. Also, no residual PbI_2_ (001) characteristic peak was observed in all three films annealed at different environments, implying complete conversion of the precursors to the perovskite under these three conditions.^50,51^ Moreover, all films consistently exhibited the highest diffraction peak at the (100) plane of the α-phase, suggesting that the (100) orientation represents the preferred crystallographic direction for perovskite crystal growth. Furthermore, the intensity of the (100) peak demonstrably increased with elevated annealing humidity, thereby suggesting enhanced crystallinity along this preferred direction under more humid conditions.

**Figure 3:**
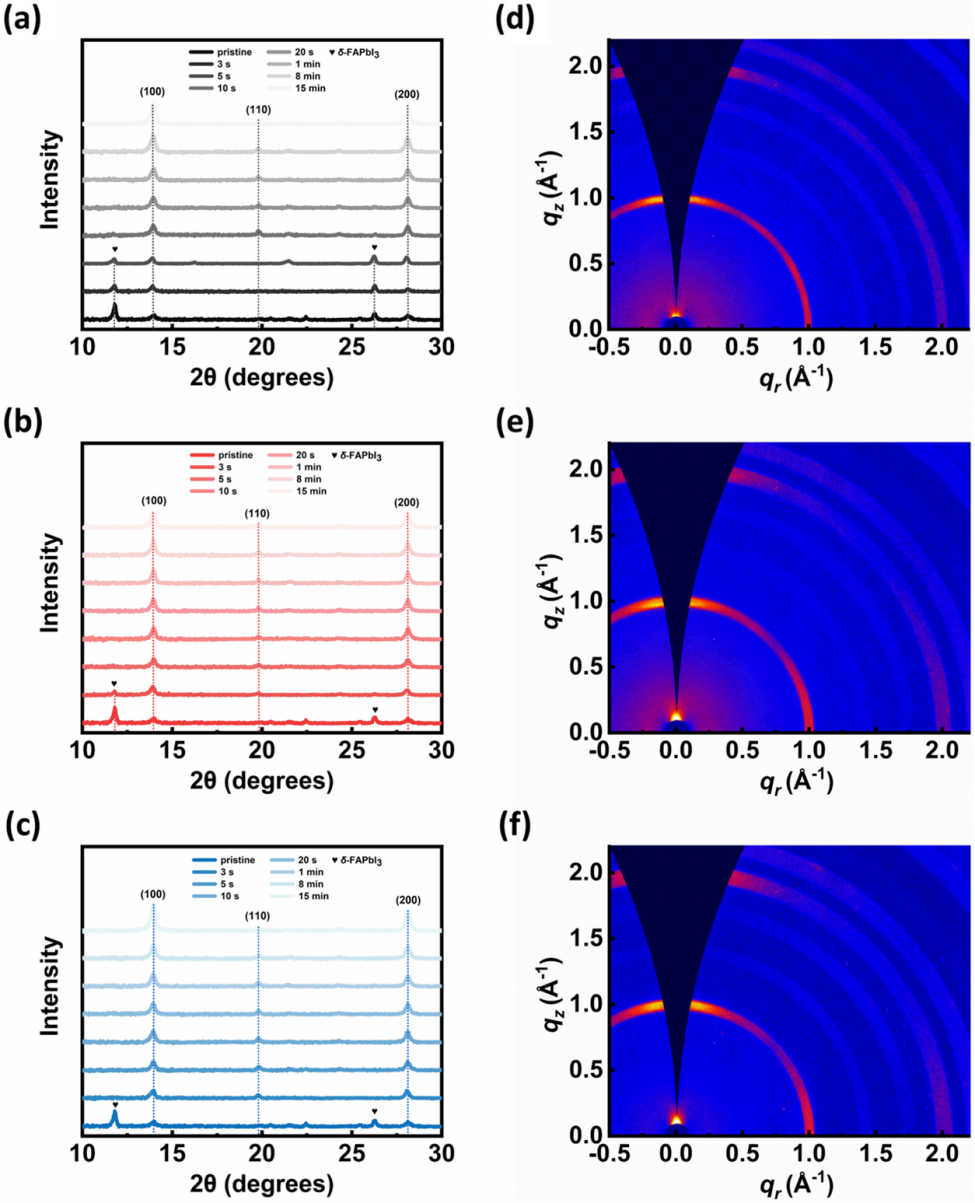
(a–c) XRD patterns of the FAPbI_3_ thin films of different annealing time under various annealing conditions. (d–f) GIWAXS analysis of the perovskite thin film annealed under different atmospheres: (a, d) N_2_, (b, e) RH 55%, and (c, f) RH 99%.

Nevertheless, a notable kinetic difference was observed in the initial phase transformation. The consumption of the δ-phase was slower in the anhydrous N_2_ atmosphere compared to the RH55% and RH99% conditions, implying distinct phase transformation dynamics. In the purely thermal process (annealing in N_2_), the δ-FAPbI_3_ to α-FAPbI_3_ transition relies solely on thermal energy to overcome the kinetic barrier for structural rearrangement. In contrast, the presence of water vapor promotes the phase transformation rate, with the transition kinetics being closely correlated with the relative humidity level. Specifically, during annealing at RH 55%, the initial δ-phase peak rapidly decayed within the first 5 s. This process was further accelerated at RH 99%, where the δ-phase signal vanished in only 3 s. The higher moisture concentration facilitated a faster complete elimination of the δ-phase, thereby ensuring a more rapid transition toward a phase-pure α-FAPbI_3_ film. Water molecules are believed to facilitate the local dissolution and rapid reorganization of the perovskite structural units, a process that may also involve the formation of transient hydrated intermediates that diminish the structural resistance for rearrangement. Consequently, this water-assisted mechanism effectively lowers the activation barrier and increases the rate of the δ-to-α phase transition.^52,53^

To further investigate the effects of different annealing conditions on the crystallinity as well as preferred orientation of the perovskite films, GIWAXS measurements were conducted on the annealed coevaporated perovskite films. The results are shown in Figure 3d–f. All perovskite thin films exhibited clear diffraction signals along both the (100) and (200) directions, which is consistent with the results obtained from our previous XRD analysis. With regard to crystal orientation, all three cases demonstrated a clear preferred orientation in both the (100) and (200) planes, with the most intense signals observed along the out-of-plane direction. Notably, the perovskite film annealed under RH 99% exhibited the strongest out-of-plane signal. A more pronounced out-of-plane orientation indicates that the grains within the film are highly ordered in the vertical direction, reflecting a preferential crystal growth along the (100) axis perpendicular to the substrate. This observation is further quantified by integrating the azimuthal intensity distributions (χ scans) of the (100) and (200) diffraction rings, as shown in Figure S8 and summarized in Table S2 (Supporting Information). Specifically, the fraction of face-on–oriented crystallites, defined as domains with the (100) planes parallel to the substrate, increases from 0.55 for the N_2_-annealed film to 0.56 under RH 55%, and reaches 0.59 for the film annealed at RH 99%, corresponding to a relative increase of approximately 7% in vertical crystallographic alignment compared to the dry annealing condition. A consistent trend is also observed for the (200) diffraction, where the face-on ratio increases from 0.50 (N_2_) to 0.53 (RH 55%) and further to 0.55 (RH 99%). The consistent enhancement across multiple diffraction orders indicates that high-humidity annealing promotes improved long-range ordering and preferential vertical alignment of the perovskite lattice. Furthermore, this structural evolution is supported by cross-sectional SEM images, which reveal vertically aligned columnar grains in the perovskite film annealed at RH 99%. The enhanced (100) out-of-plane orientation facilitates efficient vertical carrier transport while reducing recombination by minimizing horizontal grain boundaries.

To clarify the final state of the perovskite films after different annealing conditions, Fourier transform infrared spectroscopy (FTIR) was carried out. From Figure S9 (Supporting Information), All samples exhibited a characteristic absorption at 1712 cm^–1^, assigned to the C=N stretching vibration of the FA^+^ cation, along with weak N–H stretching features at approximately 3267 and 3401 cm^–1^, indicating the formation of phase-pure α-FAPbI_3_ across all annealing conditions. Notably, no broad absorption is observed in the 3000–3500 cm^–1^ region, where molecular water typically exhibits strong O–H stretching vibrations. This absence of water-related signatures indicates that no stable hydrated intermediates or residual water remain in the ∼500 nm-thick films after annealing at 170 °C for 15 min, regardless of the annealing humidity employed, supporting that water vapor acts transiently during annealing rather than being retained in the final perovskite films.^54,55^

To investigate the charge carrier transport properties of perovskite films annealed under different conditions, steady-state PL and TRPL measurements were performed on Glass/ITO/3PATAT-C3/perovskite structures. As shown in Figure S10 (Supporting Information), all films exhibited PL emission peaks near 810 nm, which is consistent with the previously measured bandgap of the material. Notably, the perovskite film annealed at RH 99% exhibited the lowest PL intensity, which may indicate more efficient charge transfer between 3PATAT-C3 and perovskite.^56^

To gain a deeper understanding of carrier transport, TRPL measurements were subsequently performed. The results, presented in Figure S11 (Supporting Information), reveal that the normalized TRPL decay is most rapid for the RH 99% sample (τ_ave_ = 3.3 ns) compared to the N_2_ (τ_ave_ = 21 ns) and RH 55% samples (τ_ave_ = 14 ns). Collectively, the PL and TRPL results imply that perovskite films annealed under RH 99% exhibit enhanced charge carrier dynamics.^57,58^ This improved efficiency of internal charge transfer is likely to facilitate better device photovoltaic performance.

We fabricated inverted-structure FAPbI_3_ PSCs, incorporating the device architecture of ITO/3PATAT-C3/perovskite/C_60_/BCP/Ag. The current density–voltage (*J*–*V*) characteristics and the corresponding external quantum efficiency (EQE) spectra of these devices are presented in Figures 4a and S12 (Supporting Information), respectively. The detailed photovoltaic performance parameters are summarized in Table 1.

**Figure 4:**
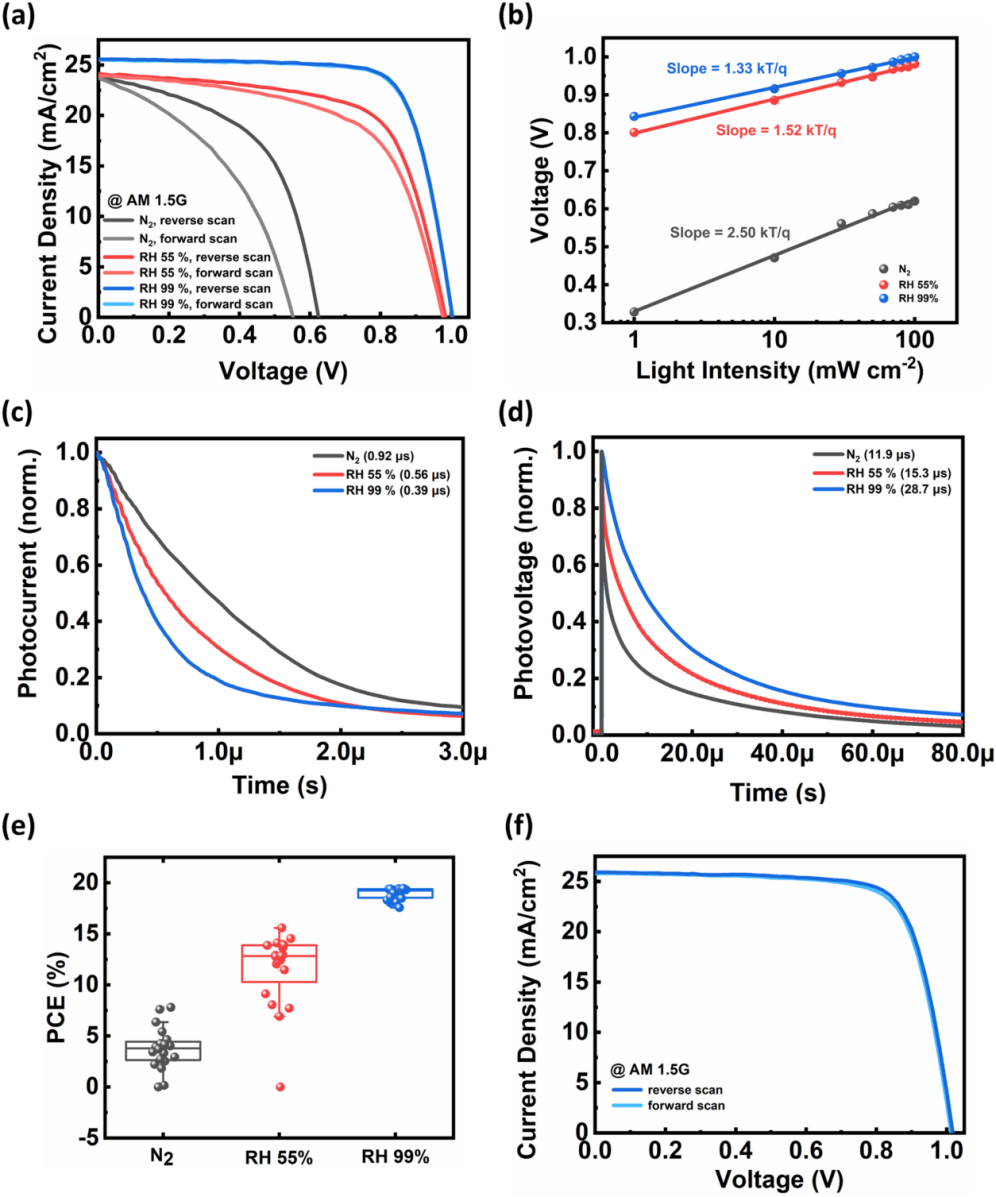
(a) Comparison of *J*–*V* characteristics of PSCs with 3PATAT-C3 as the HTL, fabricated under different annealing humidity conditions. (b) Light-intensity dependent *V*_OC_, (c) TPC and (d) TPV results of PSCs fabricated under different annealing humidity conditions. (e) Statistical distribution of PCEs for 20 PSCs fabricated under each humidity. (f) *J*–*V* characteristics of the champion device using 3PATAT-C3 as the HTL after 9 months of storage in an nitrogen-filled glovebox.

**Table 1:** Performance of FAPbI_3_ PSCs with Various Annealing Conditions Employing 3PATAT-C3 as the HTL

	*V*_oc_ [V]	*J*_SC_ [mA cm^–2^]	FF [%]	PCE [%]
N_2_-R	0.62	23.7	53.2	7.9
N_2_–F	0.55	23.7	41.6	5.4
RH 55%-R	0.98	24.1	65.6	15.6
RH 55%-F	0.98	23.9	60.4	14.1
RH 99%-R	1.00	25.6	75.8	19.5
RH 99%-F	1.00	25.5	75.4	19.3

As shown in Figure 4a, the high humidity annealing process significantly enhances the photovoltaic performance of coevaporated PSCs. The champion device, employing 3PATAT-C3 as the HTL and a perovskite active layer annealed under high-humidity conditions, exhibited a PCE of 19.5%, with a *V*_OC_ of 1.00 V, a *J*_SC_ of 25.6 mA cm^–2^, and a FF of 75.8% under AM 1.5G illumination. In contrast, devices annealed in N_2_ and at RH 55% showed inferior performance, with lower PCEs of only 7.9% and 15.6%, respectively, primarily due to reduced *V*_OC_ and FF. These performance losses could be attributed to the presence of defect states and reduced charge carrier transport efficiency.

Moreover, the annealed-at-RH 99% device exhibits a negligible hysteresis index (HI) of 0.01, which is significantly lower than that of RH 55% and N_2_ devices (0.10 and 0.32, respectively). The observed reduction in device hysteresis is attributed to microstructural evolution induced by high-humidity annealing. As evidenced by the cross-sectional SEM images and the quantitative analysis of the GIWAXS patterns shown in Figure 2f and Table S2 (Supporting Information), saturated-humidity-treated films exhibited pronounced vertical alignment, which reduces horizontally oriented grain boundaries along the transport direction. Since such grain boundaries are widely recognized as pathways for ion migration and charge trapping. Consequently, the reduced availability of such grain-boundary pathways is expected to hinder ion migration, thereby mitigating ion accumulation and charge recombination during voltage sweeps and resulting in the observed significant reduction in hysteresis.^59^ The integrated *J*_SC_ value obtained from the EQE spectrum of the RH 99% device, shown in Figure S12 (Supporting Information), is 25.4 mA cm^–2^, which is in good agreement with the *J*–*V* measurements. The steady-state photocurrent and PCE of the champion device, as measured by maximum power point (MPP) tracking, are shown in Figure S13 (Supporting Information), exhibiting a stabilized PCE of 19.4% and a steady-state current density of 23.7 mA cm^–2^.

To gain deeper insights into the impact of postannealing humidity on the trap density of the perovskite films, space-charge-limited current (SCLC) measurements were performed on electron-only devices (EODs) with the configuration ITO/SnO_2_/perovskite/C_60_/Ag, fabricated using perovskite films annealed under different humidity conditions. According to the SCLC model, the trap density (N_t_) was determined using the relation^60^

1 $$N_{t}=2\epsilon_{0}\epsilon_{r}V_{\mathrm{TFL}}/qL^{2}$$

where ϵ_0_ is the vacuum permittivity, ϵ_r_ is the relative dielectric constant of FAPbI_3_ (46.9),^61^ *V*_TFL_ is the trap-filled limit voltage, *q* is the elementary charge, and *L* is the perovskite film thickness (500 nm).

As shown in Figure S14 (Supporting Information), the trap-filled limit voltage significantly decreased from 0.547 V (N_2_) to 0.364 V (RH 55%) and 0.246 V (RH 99%). Consequently, the trap densities in the EODs decrease from 1.13 × 10^16^ cm^–3^ to 7.55 × 10^15^ cm^–3^ and 5.10 × 10^15^ cm^–3^, respectively. These results provide direct evidence that high-humidity annealing effectively suppresses bulk trap states in the perovskite layer, which is consistent with the observed improvements in device performance.

To further investigate the mechanisms of performance variations in PSCs subjected to different annealing conditions, light-intensity dependent *V*_OC_, transient photocurrent (TPC) and transient photovoltage (TPV) measurements were employed on devices with the architecture ITO/3PATAT-C3/perovskite/C_60_/BCP/Ag. The light-intensity dependent *V*_OC_ results are presented in Figure 4b. The ideality factor (*n*) was extracted from the slope of the *V*_OC_ versus logarithmic light-intensity plots, where the slope is expressed in units of kT q^–1^. A significant reduction in *n* is observed with increasing annealing humidity. Specifically, the device annealed in N_2_ exhibited a large slope of 2.50 kT q^–1^, implying pronounced nonradiative recombination induced by deep-level traps. In contrast, devices annealed at RH 55% and RH 99% showed significantly reduced slopes of approximately 1.52 and 1.33 kT q^–1^, respectively. The substantially lower ideality factor observed for the device annealed at RH 99% therefore provides quantitative evidence that high-humidity annealing effectively suppresses nonradiative recombination.^62^

As shown in Figure 4c, the device with perovskite annealed under high-humidity conditions exhibited a significantly shorter carrier transit time of 0.39 μs, in contrast to 0.92 and 0.56 μs for devices annealed in N_2_ and at RH 55%, respectively. These TPC results indicate enhanced carrier transport and higher carrier mobility for PSCs annealed at RH 99%.^8^ TPV measurements, presented in Figure 4d, reveal that as the annealing humidity increases, the carrier lifetime increases from 11.9 to 28.7 μs. Together, the TPC and TPV results demonstrate that high-humidity annealing process promotes faster carrier transport and longer carrier lifetimes. Collectively, these complementary electrical characterizations results confirm that the high-humidity annealing treatment significantly reduces trap densities, thereby extending the carrier diffusion length and effectively suppressing nonradiative recombination, and ultimately contributing to improved photovoltaic efficiency.^8,63^

The statistical distribution of photovoltaic parameters for 20 PSCs fabricated under each humidity condition, as shown in Figures 4e and S15 (Supporting Information), demonstrates that devices processed under high-humidity conditions not only yielded superior performance but also exhibited remarkable consistency, thereby showing excellent reproducibility. In contrast, devices processed under the other two annealing conditions not only exhibited lower performance but also showed less favorable performance variation, indicating poorer batch-to-batch reproducibility.

In addition to showing good process stability, it is important to highlight the long-term stability of the champion PSC. This device, which utilized 3PATAT-C3 as the HTL and was fabricated under high humidity annealing conditions (as shown in Figure 4a), was stored in a nitrogen-filled glovebox for 9 months. After this period, it still maintained excellent performance, with a *V*_OC_ of 1.02 V, a *J*_SC_ of 25.5 mA cm^–2^ (the integrated *J*_SC_ obtained from the EQE spectrum is 25.4 mA cm^–2^), a FF of 75.2%, and a PCE of 19.5%, accompanied by a negligible hysteresis index of 0.01. Further details are provided in Figures 4f and S16 (Supporting Information). The photovoltaic performance is summarized in Table S3 (Supporting Information).

For the inverted PSCs, self-assembled monolayers (SAMs) serve as critical hole-transporting materials, where the choice of SAMs significantly influences device performance.^36,64,65^ To further showcase the wide applicability of this saturated-humidity annealed perovskite active layer with various SAM underlayers, we replaced 3PATAT-C3 with Me-4PACz as the HTL in PSCs. The *J*–*V* characteristics and corresponding EQE spectra of the champion PSC are presented in Figure 5a and Figure 5b, respectively. Table 2 lists the detailed parameters of the photovoltaic performance. Remarkably, the champion device employing Me-4PACz achieved a PCE of 21.3%, clearly outperforming its 3PATAT-C3 counterparts (a PCE of 19.5%). This improvement can be mainly attributed to a significantly improved *V*_OC_ of 1.10 V, along with a *J*_SC_ of 25.9 mA cm^–2^ and a FF of 75.2% under AM 1.5G illumination. Additionally, the device also exhibited a negligible hysteresis (HI = 0.009). The corresponding EQE demonstrated a plateau curve, with a consistently high value of approximately 90% observed throughout the ultraviolet–visible-near-infrared region. The integrated *J*_SC_ value from the EQE spectrum is approximately 25.7 mA cm^–2^, which is also consistent with the *J*–*V* sweeping results. Furthermore, as presented in Figure 5c, the stabilized PCE under MPP tracking remained at 21.1% after 5 min. A significant enhancement in *V*_OC_ upon the incorporation Me-4PACz is evident when comparing the *J*–*V* characteristics of the two champion PSCs employing 3PATAT-C3 and Me-4PACz as HTLs, respectively, as shown in Figure S17 (Supporting Information).

**Figure 5:**
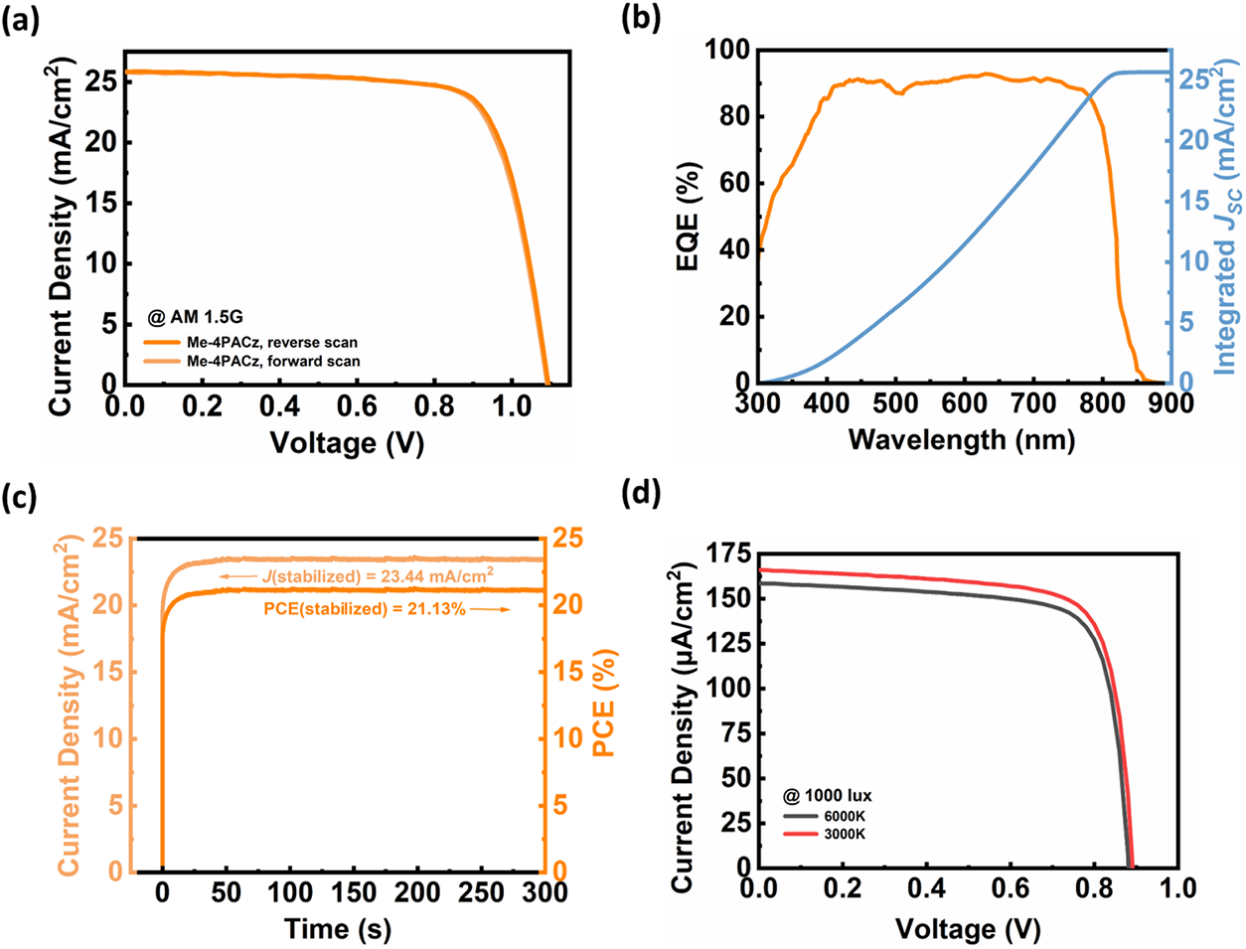
Photovoltaic performance characteristics of the champion PSC fabricated with an optimized postannealing process and employing Me-4PACz as the HTL. (a) *J*–*V* characteristics. (b) Corresponding EQE spectra with integrated *J*_SC_. (c) Stabilized photocurrent density measured under maximum power point (MPP) tracking (at 0.90 V) to demonstrate device reliability. (d) *J*–*V* characteristics measured under 6500 and 3000 K fluorescent lamp illumination at 1000 lx.

**Table 2:** Photovoltaic Parameters of the Champion Cell of FAPbI_3_ PSCs Fabricated with Optimized Post-Annealing Process Employing Me-4PACz as the HTL

	*V*_oc_ [V]	*J*_SC_ [mA cm^–2^]	FF [%]	PCE [%]
reverse scan	1.10	25.9	75.2	21.3
forward scan	1.09	25.8	74.7	21.1

To unlock the underlying mechanisms responsible for the significant enhancement in *V*_OC_, we measured the valence band of the perovskite film annealed under high-humidity annealing conditions. Ultraviolet photoelectron spectroscopy (UPS) measurements were employed, the results are shown in Figure S18 (Supporting Information). As previously noted, the perovskite exhibits a bandgap of approximately 1.53 eV. By combining this information with the UPS results, the valence band maximum (VBM) and conduction band minimum (CBM) were estimated to be 5.69 and 4.16 eV, respectively.

To investigate whether the high-vacuum environment and elevated annealing temperature required for perovskite fabrication compromise the stability and integrity of the SAM underlayer, UPS and X-ray photoelectron spectroscopy (XPS) analyses were performed on Me-4PACz under relevant processing conditions, as shown in Figures S19 and S20 (Supporting Information). UPS analysis revealed that both vacuum exposure and subsequent thermal annealing at 170 °C for 15 min result in negligible variations in the secondary electron cutoff and valence band (VB) onset. This indicates that the work-function and electronic structure of the Me-4PACz layer remain well-preserved. Consistently, XPS measurements showed no discernible changes in spectral profiles or binding energy shifts in the C 1s, N 1s, P 2p, and O 1s core-level spectra following vacuum and thermal treatments, suggesting the absence of detectable chemical decomposition or SAM desorption. These results provide evidence that neither the high-vacuum environment nor the thermal budget employed in this work compromises the chemical integrity or electronic functionality of the Me-4PACz underlayer. Notably, the observed thermal robustness aligns with literature reports indicating a decomposition temperature of approximately 348 °C for Me-4PACz, which is substantially higher than the annealing temperature used herein, thereby confirming its thermal stability for vacuum-deposited perovskite solar cells.^66^

As shown in Figure S21 (Supporting Information), Me-4PACz exhibits a deeper ionization potential of 5.59 eV, as determined from UPS measurements, compared to 3PATAT-C3 (5.44 eV). This results in a more favorable band alignment with the perovskite VBM (5.69 eV).^65^ This optimized alignment effectively suppresses energetic losses at the interface induced by energy level mismatch, while simultaneously maintaining efficient hole extraction.^67^

We also explored the potential of these coevaporated FAPbI_3_ PSCs for indoor photovoltaic applications. An illuminance of 1000 lx was employed as a benchmark intensity for professional indoor environments, serving as a universal reporting standard critical for powering the Internet of Things (IoT) ecosystem.^68^ As shown in Figure 5d and summarized in Table S4, without any changes to the device architecture, the best Me-4PACz device exhibited PCEs of 34.9% and 36.7% under 6500 K T5 and 3000 K T8 artificial fluorescent illumination of 1000 lx, respectively. The enhanced *V*_OC_ and *J*_SC_ under the lower color temperature light source can be attributed to the better spectral overlap between the 3000 K emission spectrum and the absorption range of FAPbI_3_. Specifically, lower color temperature sources emit a greater proportion of photons with energies closer to the bandgap of the perovskite absorber, thereby increasing the effective photon utilization. Although the spectral response of the FAPbI_3_ absorber is not yet fully optimized for indoor lighting conditions, the devices still deliver excellent PCEs, demonstrating that vacuum-deposited FAPbI_3_ PSCs not only achieve high efficiency under standard 1-sun illumination but also maintain low *V*_OC_ loss under dim-light conditions, which makes them highly promising for indoor energy harvesting applications. Higher PCE under narrower spectral illumination can be anticipated with bandgap engineering (e.g., Bromide incorporation) of the vacuum-deposited PSCs.

To evaluate operational stability, continuous illumination aging tests were conducted under various conditions. As shown in Figure 6a, the PSC fabricated with saturated-humidity annealing method exhibited superior stability under continuous 1-sun illumination in an N_2_ atmosphere, retaining 90.2% of its initial PCE after 500 h, whereas PSCs processed under N_2_ or RH 55% showed significantly accelerated PCE degradation. Furthermore, as illustrated in Figure 6b, the unencapsulated optimized device maintained 82.1% of its initial efficiency after 500 h under elevated temperature (65 °C) and high humidity (RH 85%). These results confirm that vacuum-deposited FAPbI_3_ PSCs incorporating the high-humidity annealing strategy exhibit exceptional operational stability under both inert and harsh environmental conditions.

**Figure 6:**
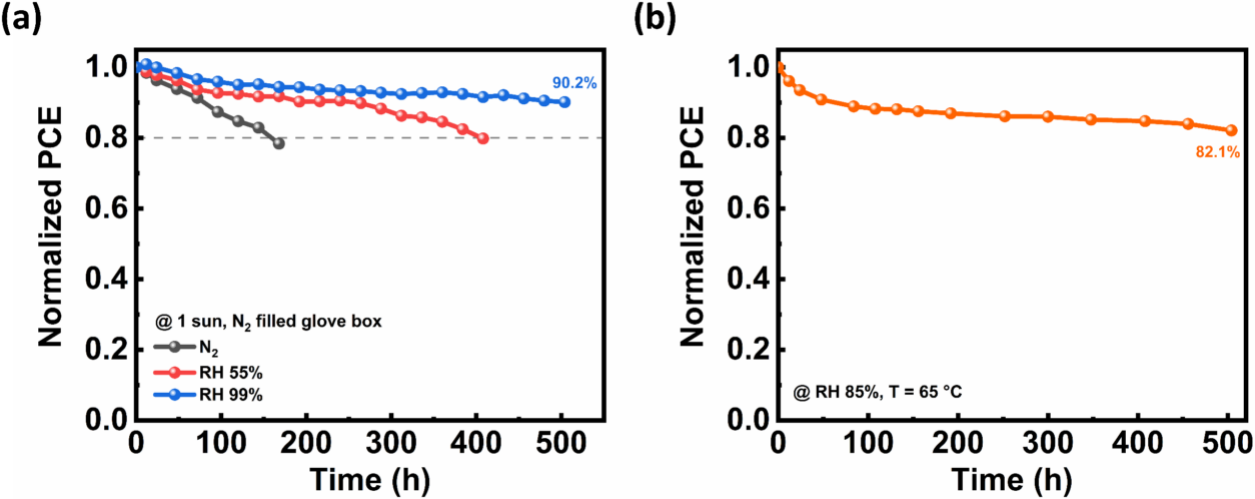
Long-term operational stability profiles of FAPbI_3_ PSCs under continuous 1-sun illumination. (a) Stability of 3PATAT-C3 based PSCs fabricated under different annealing conditions in an N_2_-filled glovebox. (b) Stability of the unencapsulated device fabricated with an optimized postannealing process and employing Me-4PACz as the HTL, evaluated at 65 °C and RH 85%.

## Conclusion

In summary, we investigated the impact of annealing humidity on the surface morphology, crystallinity, crystal orientation, density of defect states, and charge carrier transport properties of vacuum-deposited FAPbI_3_ perovskite thin films, as well as the resulting device performance. Through a saturated-humidity annealing process, we achieved pure and stable α-phase FAPbI_3_ perovskite films with superior quality. Utilizing this strategy, we fabricated inverted PSCs with PCE approaching 20% with excellent device stability and reproducibility. Further optimization of the HTL enabled a boost in efficiency to 21.3% under AM 1.5G illumination and remarkable operational stability at rigorous environments. Furthermore, the as-fabricated PSCs demonstrated promising performance under low-light conditions, delivering excellent PCE up to 36.7%. Overall, our work provides critical insights into the impact of annealing atmosphere on perovskite film quality and represents the first examples of PSCs capable of undergoing postannealing in a superhumid environment. This approach is much easier to achieve than the dedicated low-humidity (usually ≤30%) process space usually required, thereby paving the way for the development of high-efficiency inverted FAPbI_3_ PSCs using industrial-compatible and promising processes.

## Methods

### Substrates Preparation

ITO glass substrates (1.9 cm × 1.9 cm) were sequentially cleaned in ultrasonic bath using industrial-grade acetone, detergent, deionized water, and electronic-grade acetone for 15 min sequentially. Subsequent cleaning was performed by immersing the substrates in boiling acetone and boiling methanol, each for 15 min, followed by ultraviolet–ozone (UVO) treatment for 10 min.

### Hole-Transporting Layer Preparation

The 3PATAT-C3 solution (0.1 mM in DMF) was spin-coated onto UVO-treated ITO substrates at 3000 rpm for 30 s, followed by annealing at 110 °C for 10 min. A 0.33 mg mL^–1^ solution of Me-4PACz was prepared by dissolving the powder in IPA. Subsequently, SAMs were formed through spin-coating the Me-4PACz solution onto UVO-treated ITO substrates at 3000 rpm for 30 s, followed by annealing at 100 °C for 10 min. Residual molecules on the substrate surface were subsequently removed by rinsing with 150 μL of IPA via spin-coating at 3000 rpm for 30 s. The rinsed substrates were then annealed again at 100 °C for 10 min. All these annealing processes mentioned above were carried out inside a nitrogen-filled glovebox.

### Device Fabrication

1500 mg of PbI_2_ and 600 mg of FAI were loaded into separate crucibles and then placed into a high-vacuum thermal evaporation chamber. The FAI powder was pretreated in a nitrogen-filled glovebox by heating at 150 °C for 20 min to remove adsorbed moisture to prevent outgassing during deposition. Substrates precoated with SAMs were then loaded into the chamber (base pressure <2 × 10^–6^ Torr) to perform dual-source coevaporation. PbI_2_ and FAI were initiated with a molar ratio of 1:1. Deposition rates were controlled at 0.6 Å/s for PbI_2_ and 0.2 Å/s for FAI, and monitored by traditional quartz crystal monitors (QCM) positioned near both the sources and the substrate holder. Deposition rates were maintained constant throughout the process. The codeposition process was terminated when the PbI_2_ thickness reached 250 nm. After deposition, the chamber was infiltrating with nitrogen to retrieve the as-deposited films. Subsequently, the thin films were annealed at 170 °C for 15 min. Notably, the key process to obtain high-quality α-FAPbI_3_ perovskite thin films was annealing at RH 99%. To establish an RH 99% environment for postannealing, two beakers each containing 150 mL of deionized water were placed on a hot plate and heated to 170 °C inside a fully enclosed apparatus. The perovskite films were introduced into the apparatus after the humidity sensor reached a stable equilibrium of RH 99%. The apparatus remained sealed throughout the annealing process to ensure that the humidity level was consistently maintained. After annealing, the samples were transferred to another vacuum chamber (base pressure <2 × 10^–6^ Torr) for sequential deposition of C_60_, BCP, and Ag. 30 nm of C_60_ and 8 nm of BCP were deposited at rates of 0.5 Å/s and 0.3 Å/s, respectively. Finally, 140 nm of Ag was deposited at a rate of 6.0 Å/s. The devices were configured as follows: Glass/ITO (140 nm)/3PATAT-C3 or Me-4PACz/perovskite/C_60_ (30 nm)/BCP (8 nm)/Ag (140 nm). Following fabrication, the devices were encapsulated under an anhydrous nitrogen atmosphere using a UV-curable sealant (*Everwide Chemical Co., Epowide EX*) and a cover glass, and subsequently characterized under ambient conditions.

### Characterizations

SEM images were acquired using a *JSM-IT800* schottky field emission scanning electron microscope. Grain-size distributions were obtained by analyzing the SEM micrographs with a custom-developed program. The AFM images were obtained using a *Bruker Dimension Icon* atomic force microscope. The absorption spectra were measured by a *SHIMADZU UV-2600* UV–vis spectrophotometer. The X-ray diffraction were performed using a *Bruker D8 Advance* equipped with Cu Kα radiation. The GIWAXS measurements were performed at beamline 23A1 (BL 23A1) of the National Synchrotron Radiation Research Center (NSRRC) in Taiwan. Monochromatic X-rays with a wavelength of 1.2398 Å were used at an incident angle of 0.2° for the measurements. Samples were characterized using a custom-modified confocal microscope (South-Port Co.) equipped with an air objective lens (Zeiss; NA = 0.75) and a 405 nm pulsed diode laser (LDH–P-C-405B, PicoQuant) as the excitation source, delivering a spot size of approximately 660 nm. TRPL measurements were performed using an avalanche photodiode (SPCM-AQRH-16, Excelitas) placed behind a long-pass filter to selectively collect the emitted photons. The photon arrival times were recorded and accumulated using a time-correlated single-photon counting (TCSPC) module (HydraHarp 400, PicoQuant). Steady-state PL spectra were obtained by directing the collected photons to a spectrometer integrated with an intensified charge-coupled device (ICCD) camera. FTIR spectra were recorded using a *Bruker Vertex 80v* spectrometer. XPS and UPS measurements were performed using an electron spectroscopy for chemical analysis (ULVAC-PHI, PHI 5000 VersaProbe II). The UPS measurements, valence band UV photoemission spectra were obtained using HeI (21.22 eV) as the excitation source. A sample bias of – 5 eV was applied when acquiring the secondary electron cutoff spectra. The device current density–voltage (*J*–*V*) characteristics were measured using a Semiconductor Analyzer (*B1500, Keysight*) in the dark and under AM 1.5G simulated solar illumination with an intensity of 100 mW/cm^2^ (1 sun), calibrated by a NREL-traceable KG5 filtered silicon reference cell. The device areas (0.051 cm^2^) were defined by shadow masks. The EQE spectra were measured by illuminating the PSCs under a chopped monochromatic light with a continuous-wave bias white light (from a halogen lamp). The monochromatic light intensities were calibrated using a NIST-traceable power meter (*Ophir*). The photocurrent signals were then extracted by using a current preamplifier (*Stanford Research System*) followed by a lock-in amplifier (*SR860*). The indoor *J*–V curves were measured under 6500K and 3000K fluorescent light sources. The SCLC and Light-intensity dependent *V*_OC_ measurements were performed via a sourcemeter (Keithley 2600 B). A custom-built system was constructed to perform TPC and TPV measurements. Laser pulses (405 nm) were used as the triggering source, with a pulse width of 5 ns and a repetition frequency of 10 Hz.

## Supplementary Material



## Notes

Photograph of the apparatus for postannealing; the photograpth of large-area perovskite thin film and large-area PSC; top-view SEM images of the pristine film; grain size distribution histograms extracted from SEM images of perovskite films annealed under various conditions; 3D AFM surface roughness images of the perovskite annealed under different humidity conditions; optical properties and bandgap determination from the absorption spectea of perovskite thin films annealed under different conditions; Azimuthal intensity plots of FAPbI_3_ thin films extracted along the (100) and (200) diffraction rings; FTIR spectra of perovskite thin films annealed under different conditions; steady-state PL spectra and TRPL results of perovskite films with a 3PATAT-C3 hole-transporting underlayer; the corresponding EQE spectra of PSCs with 3PATAT-C3 as the HTL; stabilized photocurrent density measured under maximum power point of the champion PSC annealed at RH 99% and employing 3PATAT-C3 as the HTL; SCLC plots of the electron-only devices fabricated under different annealing humidity conditions; statistical distribution of photovoltaic parameters for 20 PSCs fabricated under each humidity condition; corresponding EQE spectra of the champion device using 3PATAT-C3 as the HTL after 9 months of storage in a nitrogen-filled glovebox; the comparison of *J*–*V* characteristics between 2 champion devices applying different HTLs. UPS spectrum of the perovskite film; UPS and XPS results of Me-4PACz samples simulating different fabrication stages and bare ITO; device energy level diagram; tables of quantitative summary of grain size statistics and crystallographic orientation for perovskite films annealed under different humidity conditions; tables of detailed photovoltaic parameters of PSCs (PDF)
