# Supplementary material for: Realizing High-Performance Vacuum-Deposited Inverted α‑FAPbI3 Perovskite Solar Cells through Saturated-Humidity Annealing
Source: ACS Appl Mater Interfaces. 2026 Mar 18;18(12):18049–61. doi: 10.1021/acsami.5c24183 (PMC13051464; doi:10.1021/acsami.5c24183)
Supplement: Supplementary file 1 [file am5c24183_si_001.pdf]

Supporting Information

# Realizing High-Performance Vacuum-Deposited Inverted $\alpha$-$FAPbI_3$ Perovskite Solar Cells Through Saturated-Humidity Annealing

*Yun-Sheng Jheng[a], Cheng-Yueh Chen[a], Pei-En Jan[a], Hung-Ming Chen[a], Hao-Cheng Lin[a], Ping-Hsun Tsai[a], Chia-Feng Li[b], Yu-Ching Huang[b], Minh Anh Truong[c], Atsushi Wakamiya[c] and Hao-Wu Lin*[a,d]*

a. Department of Materials Science and Engineering, National Tsing Hua University, Hsinchu 30013, Taiwan

b. Department of Materials Engineering, Ming Chi University of Technology, New Taipei City 24301, Taiwan

c. Institute for Chemical Research, Kyoto University, Gokasho, Uji, Kyoto, 611-0011, Japan.

d. Research Center for Critical Issues, Academia Sinica, Tainan 711, Taiwan.

*. To whom correspondence should be addressed.

E-mail: hwlin@mx.nthu.edu.tw (H.-W. Lin)

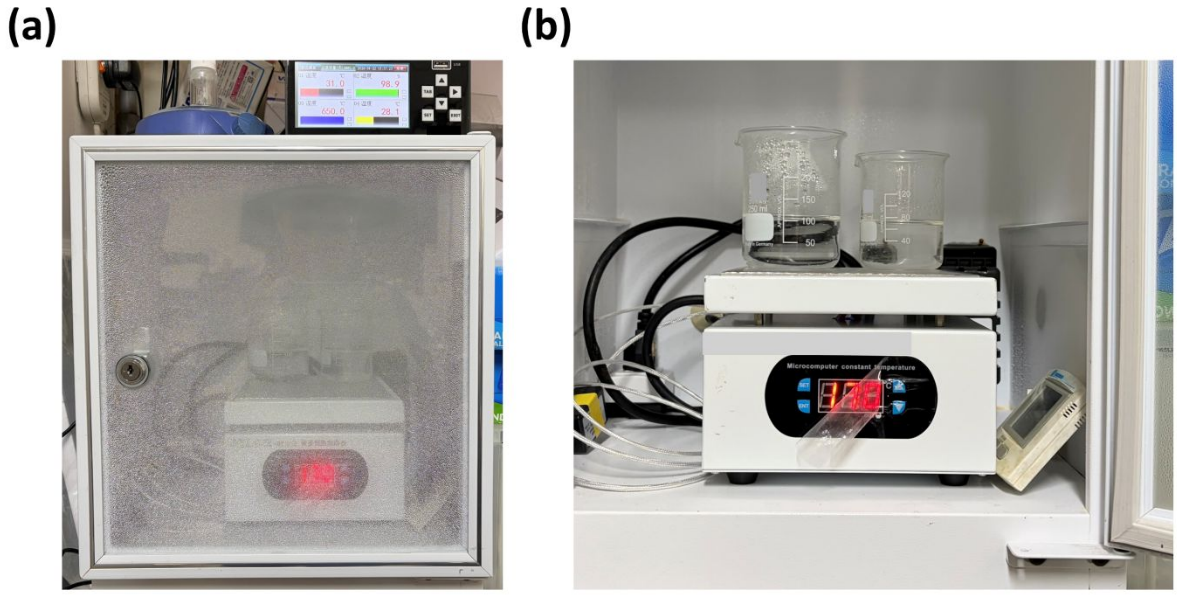


**Figure S1.** Experimental setup and verification of the high-humidity annealing environment. a) Photograph of the sealed apparatus reaching an equilibrium of RH ~99% prior to film annealing. b) Internal view of the chamber captured immediately after the annealing process. The distinct water condensation on the interior walls and beakers provides visual confirmation that a saturated water-vapor environment was maintained throughout the annealing process.

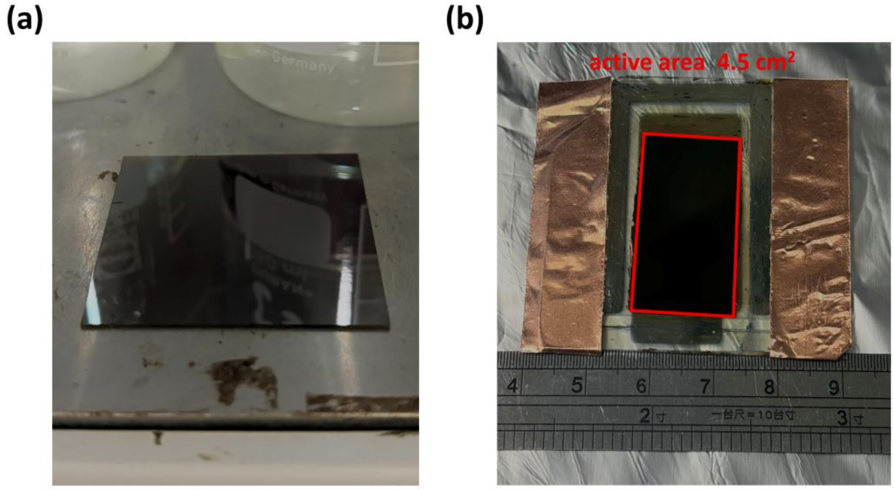


**Figure S2.** a) The photograpth of large-area perovskite thin film (25 cm$^2$) annealed using the saturated-humidity process. b) The photograpth of the device with an active area of 4.5 cm$^2$.

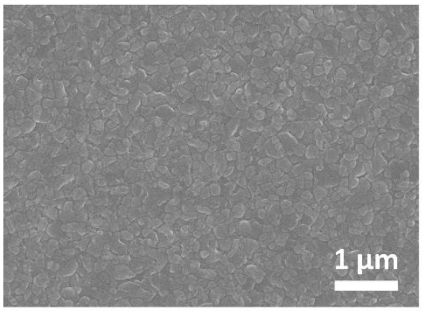


**Figure S3.** Top-view SEM images of the pristine film.

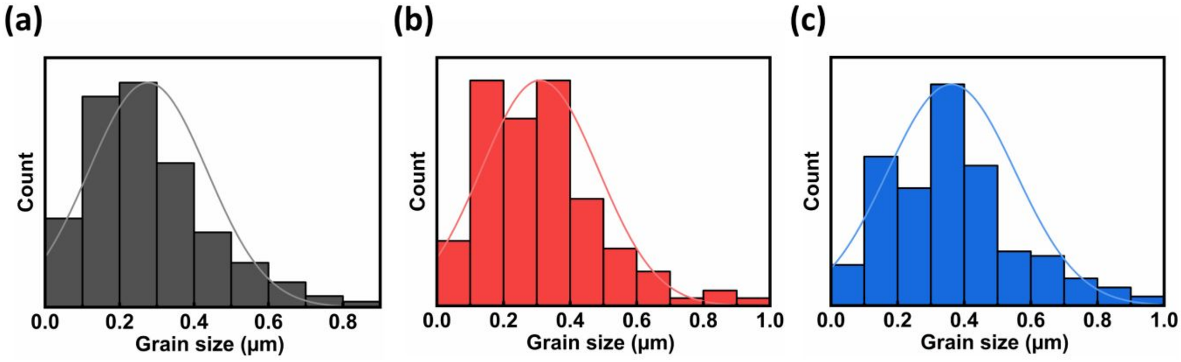


**Figure S4.** Corresponding grain size distributions extracted from the SEM images: a) $N_2$, b) RH 55%, and c) RH 99%.

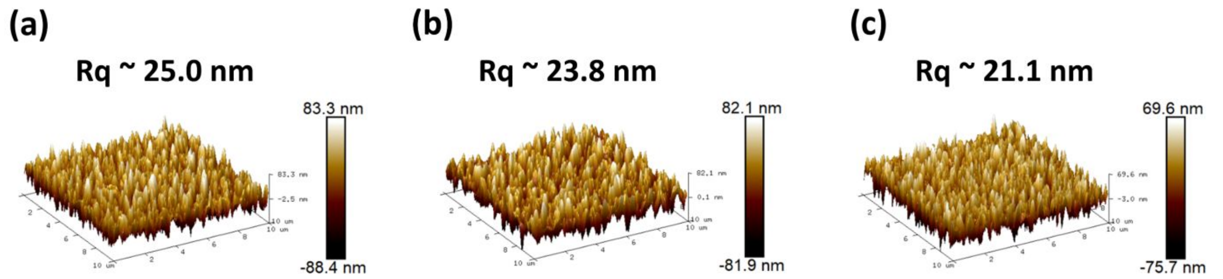


**Figure S5.** 3D AFM surface roughness images of the perovskite annealed under a) $N_2$, b) RH 55% and c) RH 99% environments.

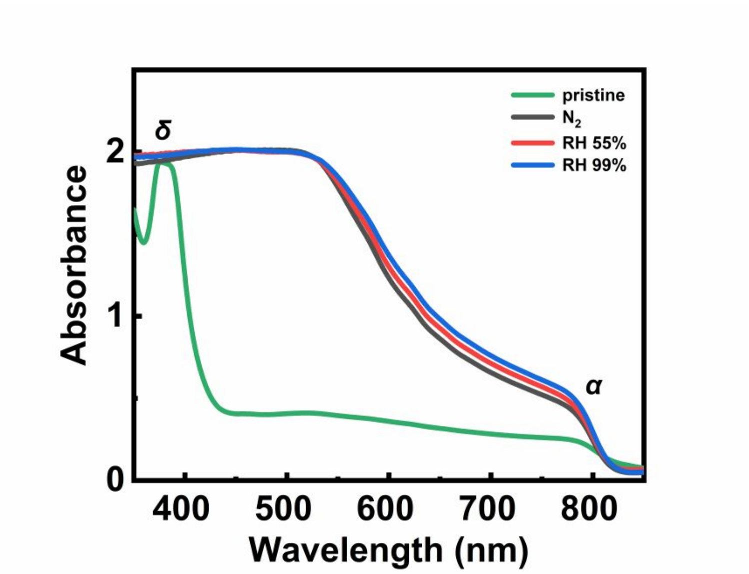


**Figure S6.** Absorption spectra of the pristine sample and 500-nm perovskite thin films annealed under different conditions.

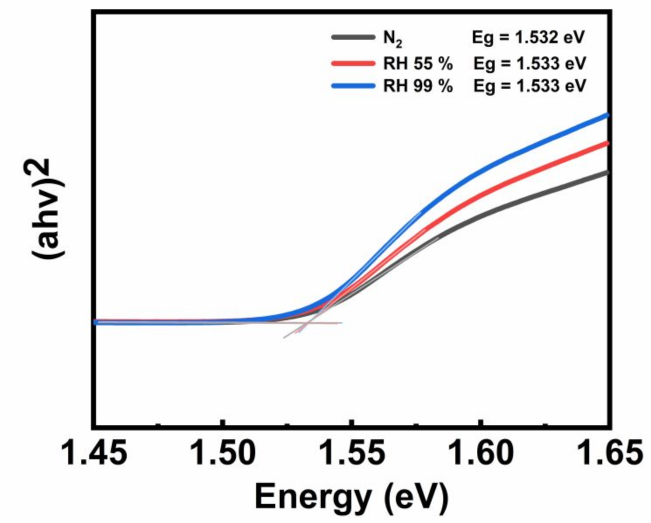


**Figure S7.** Bandgap derivation from the absorption spectea of perovskite thin films annealed under different humidity environments.

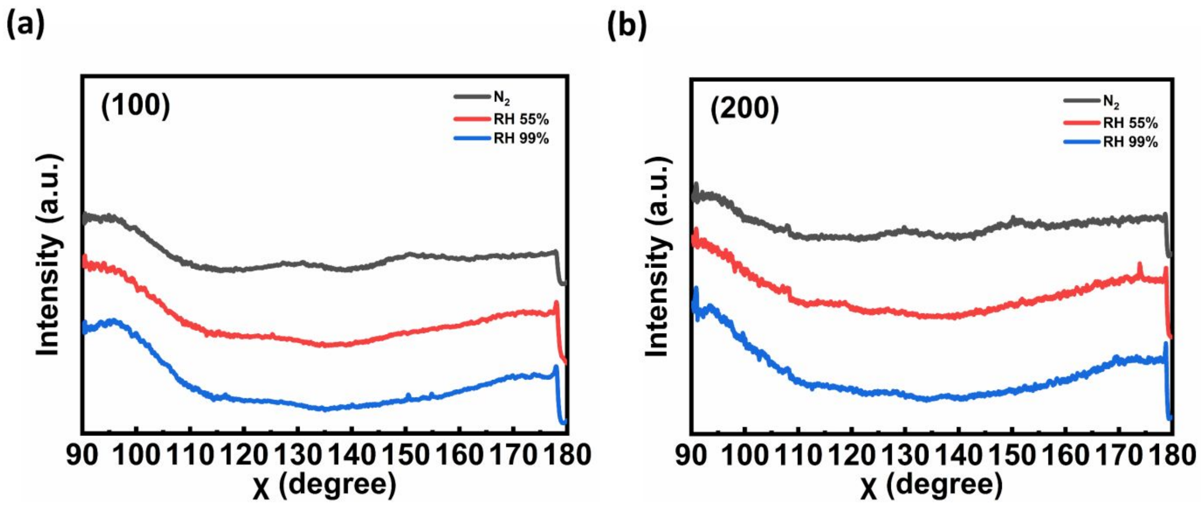


**Figure S8.** Azimuthal intensity plots (χ scans) of $FAPbI_3$ thin films extracted along the a) (100) and b) (200) diffraction rings. The films were annealed under different conditions. The peaks centered at 90° indicate a preferred out-of-plane orientation perpendicular to the substrate.

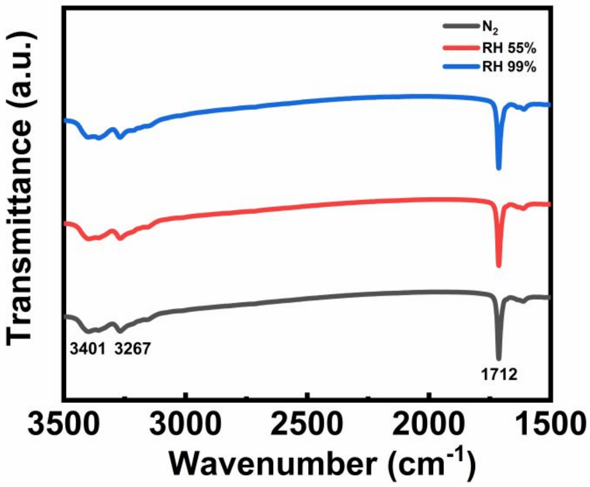

**Figure S9.** FTIR spectra of 500 nm perovskite thin films annealed under different conditions.

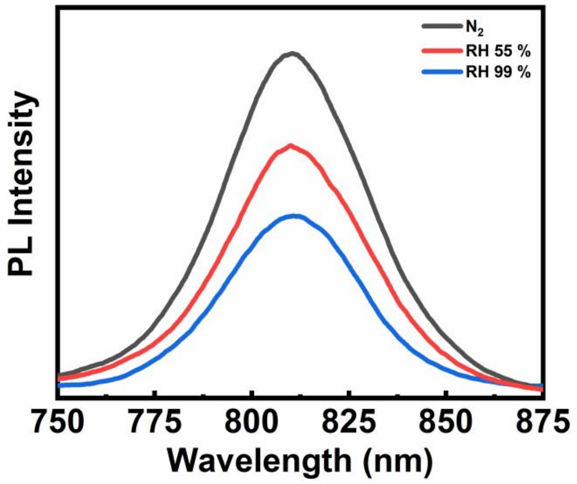


**Figure S10.** Steady-state PL spectra of perovskite films with a 3PATAT-C3 hole-transporting underlayer.

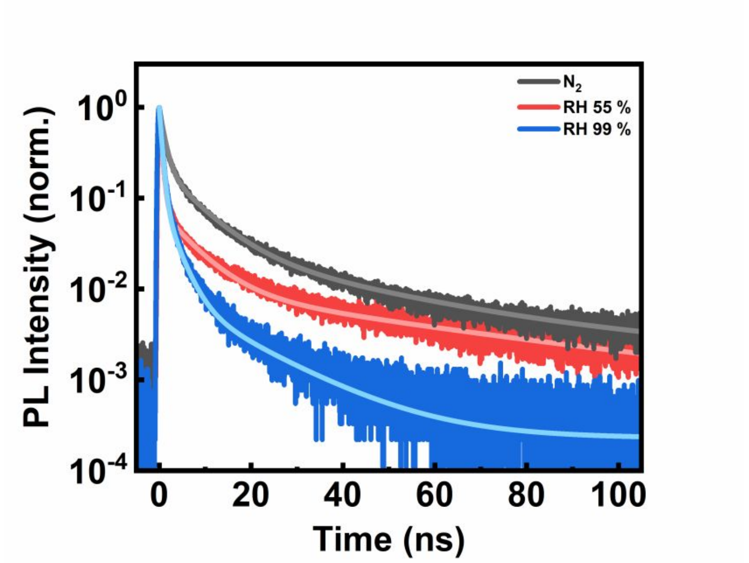


**Figure S11.** TRPL results of perovskite films with a 3PATAT-C3 hole-transporting underlayer.

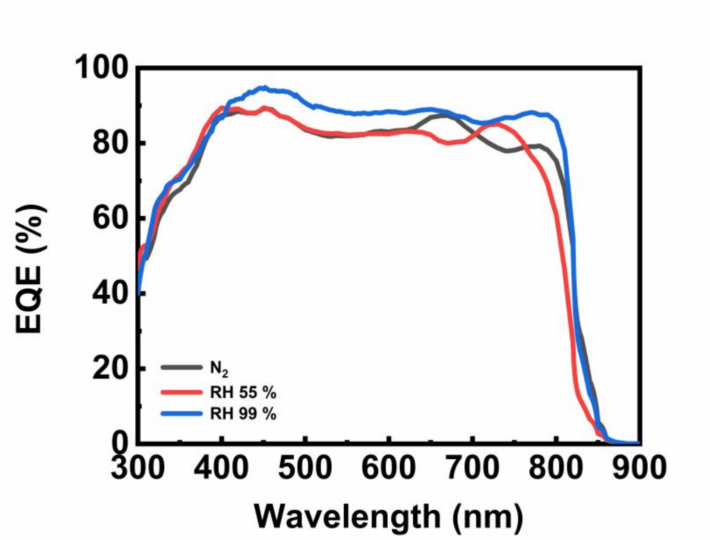


**Figure S12.** The corresponding EQE spectra of PSCs with 3PATAT-C3 as the HTL, fabricated under different annealing humidity conditions. The integrated $J_{SC}$ values from the EQE spectra are 24.0 mA cm$^{-2}$, 23.4 mA cm$^{-2}$, 25.4 mA cm$^{-2}$ for devices annealed in $N_2$, at RH 55% and at RH 99%, respectively.

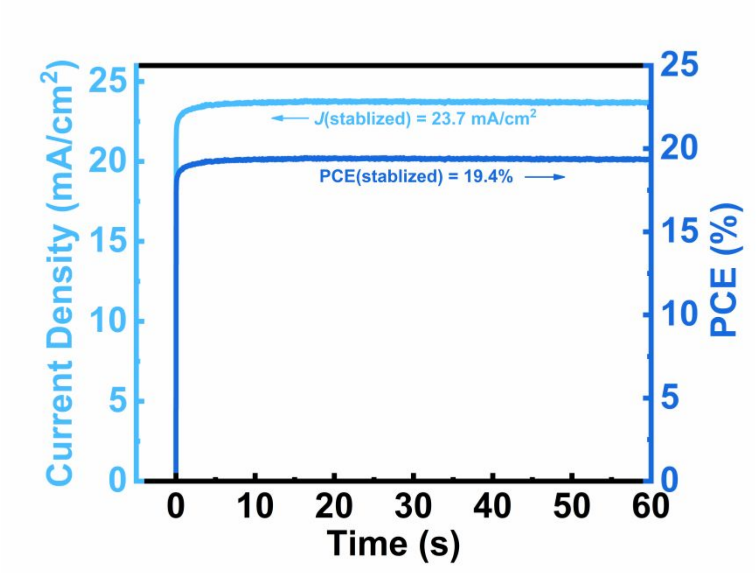


**Figure S13.** Stabilized photocurrent density measured under maximum power point (0.82 V) to demonstrate the reliability performance of the champion PSC annealed at RH 99% and employing 3PATAT-C3 as the HTL, as presented in Figure 4a.

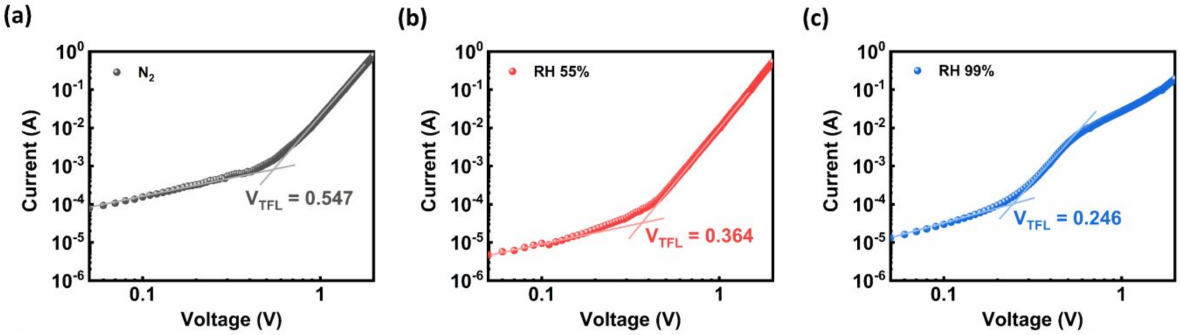


**Figure S14.** SCLC plots of the electron-only devices fabricated under a) $N_2$, b) RH 55% and c) RH 99%.

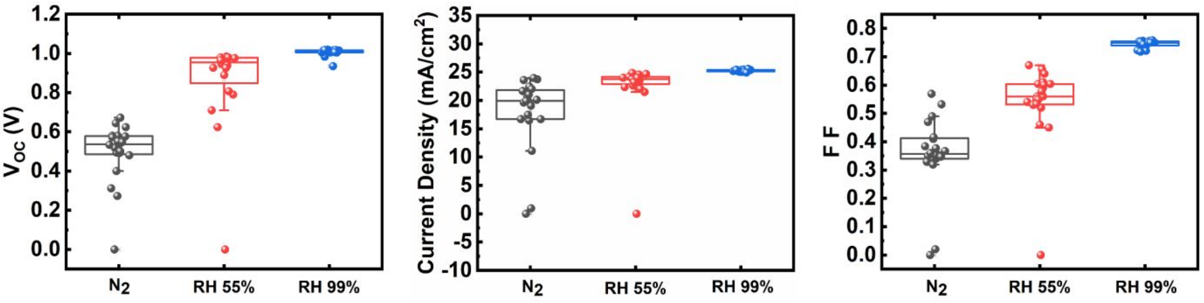


**Figure S15.** Statistical distribution of photovoltaic parameters (Voc, *Jsc* and FF) for 20 PSCs fabricated under each humidity condition, corresponding to Figure 4e.

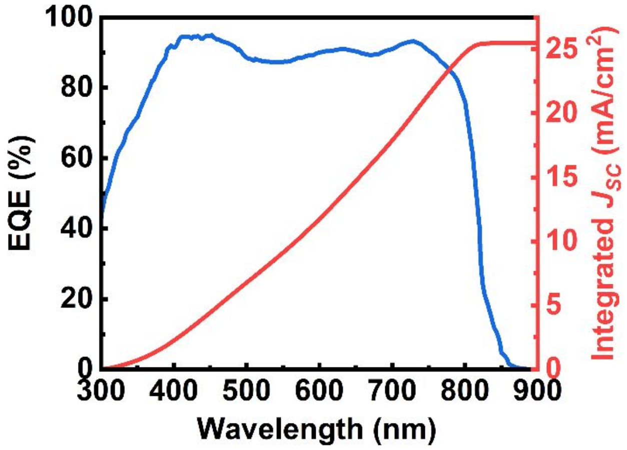


**Figure S16.** Corresponding EQE spectra (with an integrated $J_{SC}$ value of 25.4 mA cm$^{-2}$) of the champion device using 3PATAT-C3 as the HTL after 9 months of storage in a nitrogen-filled glovebox to show the long-term stability of $FAPbI_3$ PSCs fabricated under a RH 99% post-annealing strategy.

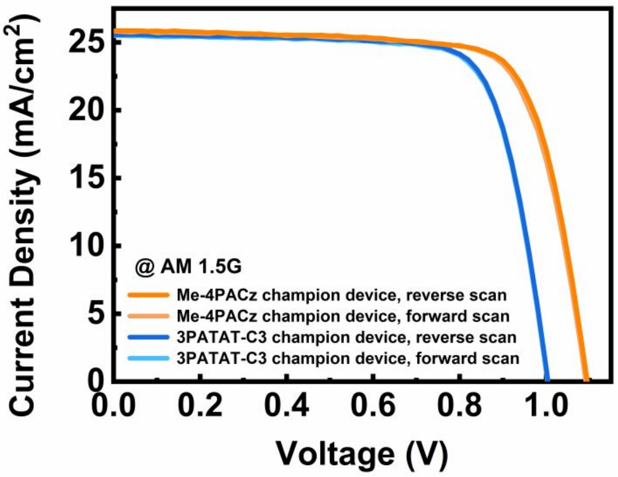


**Figure S17.** The comparison of *J*–V characteristics between 2 champion devices applying different HTLs.

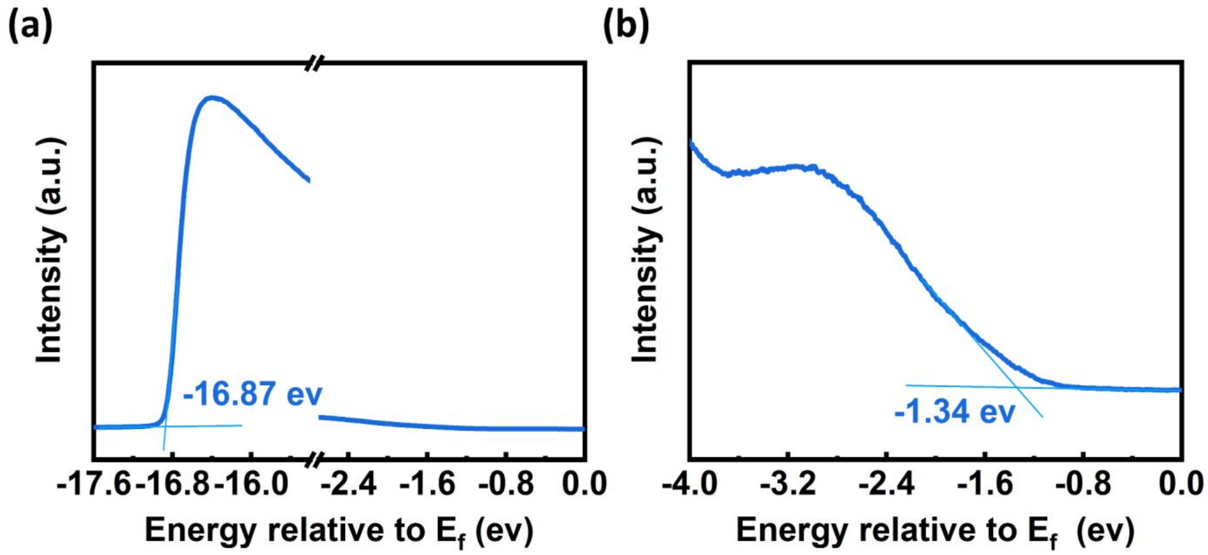


**Figure S18.** Ultraviolet photoelectron spectroscopy (UPS) spectrum of the perovskite film: a) secondary electron cutoff region used to determine the work function and b) valence band region used to determine the HOMO level. The onset on the low binding energy side corresponds to the transition of the work function from the vacuum level to the Fermi level, while the high binding energy onset represents the peak of the highest occupied molecular orbital (HOMO), indicating the energy position of the HOMO level. The work function (WF) is determined using the equation WF = hν − Eonset, where hν refers to the photon energy of He I radiation (21.22 eV), and Eonset denotes the binding energy associated with the secondary electron cutoff. The onset at the HOMO region reflects the energy difference between the HOMO level and the Fermi level. These two parameters derived from the spectrum enable accurate construction of the energy band alignment of the device architecture, as schematically illustrated in Figure S21.

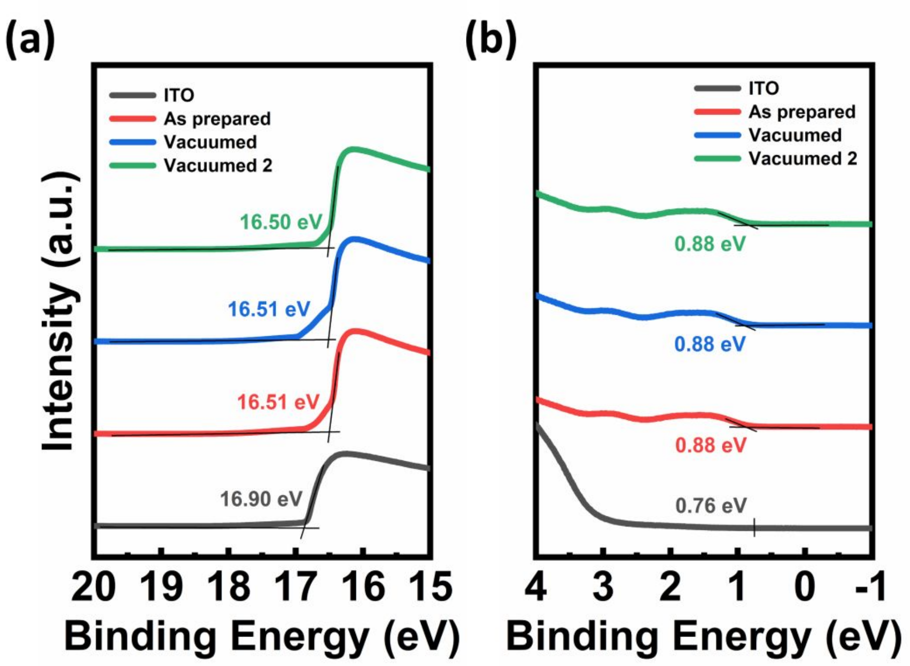


**Figure S19.** UPS results of bare ITO, the Me-4PACz sample prior to vacuum exposure (denoted as "as-prepared"), the Me-4PACz sample maintained in the vacuum chamber for a period comparable to the evaporation process (denoted as "vacuumed") and the Me-4PACz sample that was first exposed to the vacuum environment and subsequently subjected to a thermal treatment at 170 °C for 15 min (denoted as "vacuumed 2"), showing a) the secondary electron cutoff region and b) the valence band (VB) onset.

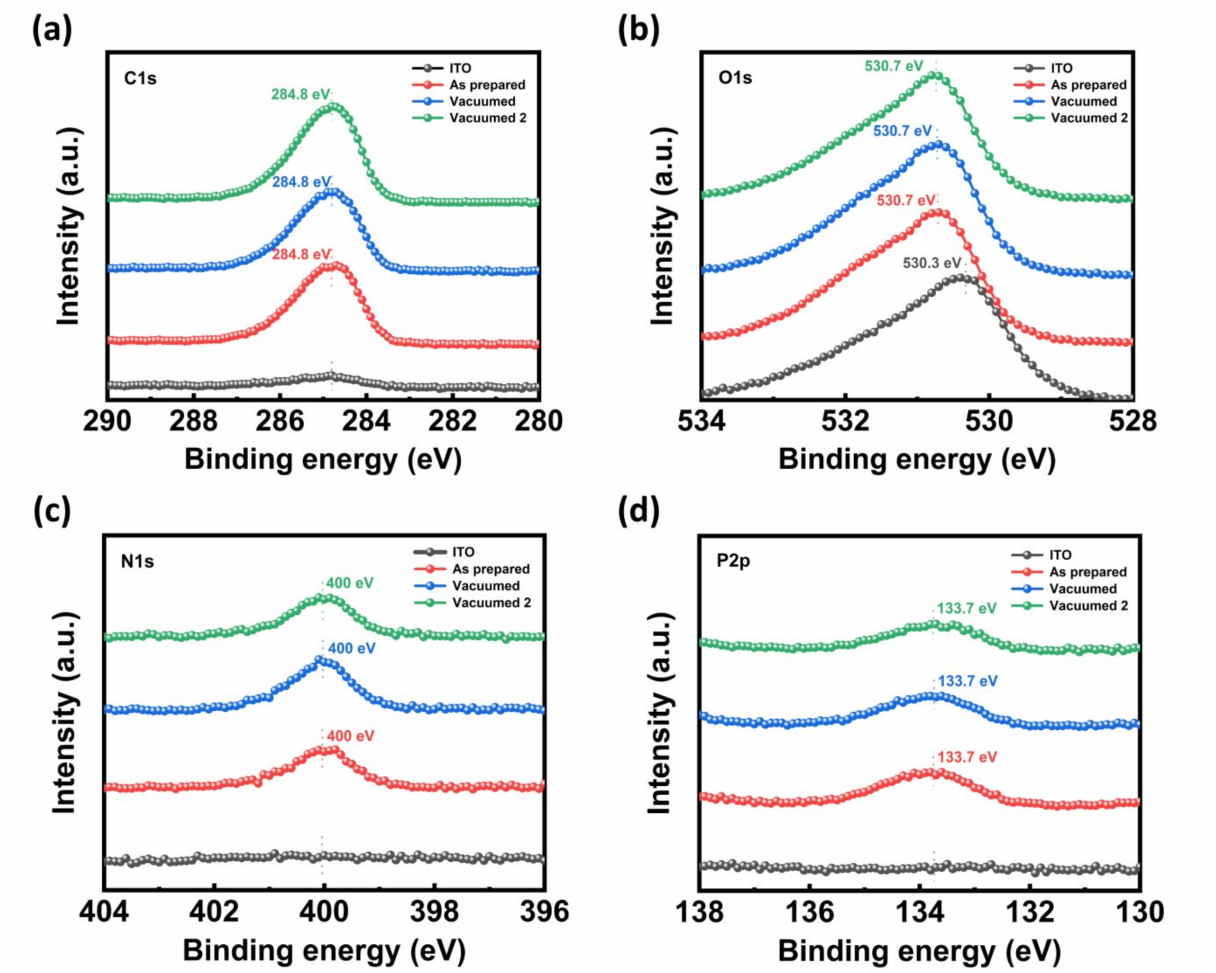


**Figure S20.** X-ray photoelectron spectroscopy (XPS) results of a) C 1s, b) O 1s, c) N 1s and d) P 2p of the samples corresponding to those shown in Figure S19.

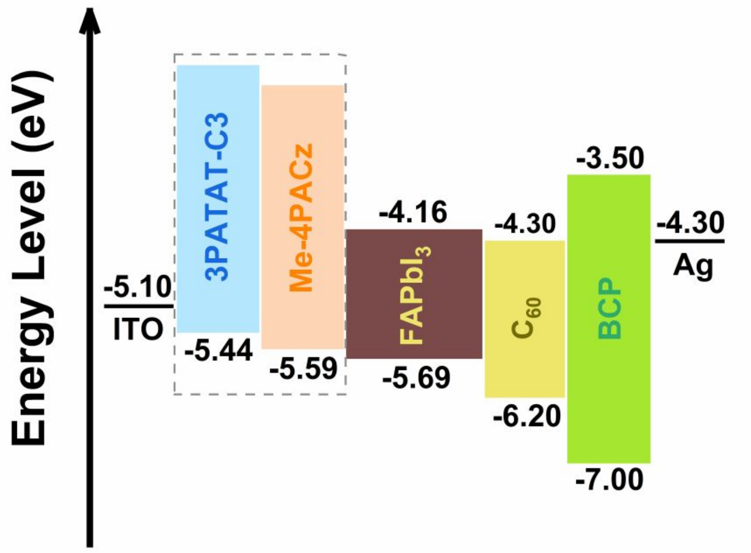


**Figure S21.** Schematic illustration of the energy level diagram of the devices employed in this work.

**Table S1.** Quantitative summary of grain size statistics for perovskite films annealed under different humidity conditions.

| Annealing Conditions | Average grain size (μm) | Coefficient of variation |
|---|---|---|
| $N_2$ | 0.28 | 0.58 |
| RH 55% | 0.31 | 0.56 |
| RH 99% | 0.36 | 0.53 |

**Table S2.** Quantitative summary of crystallographic orientation for $FAPbI_3$ films annealed under different humidity conditions. The "Face-on" and "Edge-on" ratios were calculated by integrating the azimuthal intensity distributions of the (100) and (200) diffraction rings.

| Annealing Conditions | (100) | | (200) | |
|---|---|---|---|---|
| | Face on (%) | Edge on (%) | Face on (%) | Edge on (%) |
| $N_2$ | 0.55 | 0.45 | 0.50 | 0.50 |
| RH 55% | 0.56 | 0.44 | 0.53 | 0.47 |
| RH 99% | 0.59 | 0.41 | 0.55 | 0.45 |

**Table S3.** Photovoltaic parameters of the champion cell of PSCs using 3PATAT-C3 as the HTL after 9 months of storage in a nitrogen-filled glovebox

| | **Voc [V]** | **$J_{SC}$ [mA cm$^{-2}$]** | **FF [%]** | **PCE [%]** |
|---|---|---|---|---|
| Reverse scan | 1.02 | 25.5 | 75.2 | 19.5 |
| Forward scan | 1.01 | 25.4 | 75.1 | 19.3 |

**Table S4.** Photovoltaic parameters of the champion device of PSCs fabricated with optimized post-annealing process employing Me-4PACz as the HTL measured under various fluorescent lamp illumination of 1000 lux.

| | **Voc [V]** | **$J_{SC}$ [μA cm$^{-2}$]** | **FF [%]** | **PCE [%]** |
|---|---|---|---|---|
| 6500K T5 | 0.88 | 158.7 | 75.6 | 34.9 |
| 3000K T8 | 0.89 | 166.2 | 75.2 | 36.7 |
